# 

**Published:** 1898-01

**Authors:** 


### CONTENTS.

COMMUNICATIONS.

Bridge-Work ...... 1
The Preliminary Education of the Dental Student ...... 11
Ethics—Professional...... 14
Does the Present Method of Teaching by Didactic Lectures Best Qualify the Student for the Practice of His Profession?...... 19
Abstract of Report and Clinics of the Southern Dental Association,... 22
Abstract of Report of Committee on Appliances and Improvements... 24
Silver Nitrate--Its Use in Dentistry ...... 25
Treatment of Pulpless Teeth...... 27
Practical Management of Infants...... 35

EDITORIAL

The "Register"...... 49

NOTICES.

University of Tennessee, Dental Department, Nashville, Tenn., Alumni Clinic ...... 50

